# Ultra-dense Motion Capture: An exploratory full-automatic approach for dense tracking of breast motion in 4D

**DOI:** 10.1371/journal.pone.0299040

**Published:** 2024-02-26

**Authors:** Qi-long Liu, Kit-lun Yick, Yue Sun, Joanne Yip

**Affiliations:** 1 School of Fashion and Textiles, The Hong Kong Polytechnic University, Hong Kong, China; 2 Laboratory for Artificial Intelligence in Design, Hong Kong, China; 3 School of Fashion Design & Engineering, Zhejiang Sci-Tech University, Hangzhou, Zhejiang, China; Purdue University, UNITED STATES

## Abstract

Understanding the dynamic deformation pattern and biomechanical properties of breasts is crucial in various fields, including designing ergonomic bras and customized prostheses, as well as in clinical practice. Previous studies have recorded and analyzed the dynamic behaviors of the breast surface using 4D scanning, which provides a sequence of 3D meshes during movement with high spatial and temporal resolutions. However, these studies are limited by the lack of robust and automated data processing methods which result in limited data coverage or error-prone analysis results. To address this issue, we identify revealing inter-frame dense correspondence as the core challenge towards conducting reliable and consistent analysis of the 4D scanning data. We proposed a fully-automatic approach named Ulta-dense Motion Capture (UdMC) using Thin-plate Spline (TPS) to augment the sparse landmarks recorded via motion capture (MoCap) as initial dense correspondence and then rectified it with a sophisticated post-alignment scheme. Two downstream tasks are demonstrated to validate its applicability: virtual landmark tracking and deformation intensity analysis. For evaluation, a dynamic 4D human breast anthropometric dataset *DynaBreastLite* was constructed. The results show that our approach can robustly capture the dynamic deformation characteristics of the breast surfaces, significantly outperforms baselines adapted from previous works in terms of accuracy, consistency, and efficiency. For 10 fps dataset, average error of 0.25 cm on control-landmarks and 0.33 cm on non-control (arbitrary) landmarks were achieved, with 17-70 times faster computation time. Evaluation was also carried out on 60 fps and 120 fps datasets, with consistent and large performance gaining being observed. The proposed method may contribute to advancing research in breast anthropometry, biomechanics, and ergonomics by enabling more accurate tracking of the breast surface deformation patterns and dynamic characteristics.

## Introduction

The human breast is a complex organ that undergoes significant deformations during exercise and daily activities, which can cause discomfort or even injury [1]. Sports bras have been developed to protect the breast tissues from deformation [2–4], e.g. reducing bouncing, sagging, and swinging. However, sports bras exert a high degree of pressure onto the wearer, which may have adverse effects [5]. To develop sports bras that effectively limiting breast movement while minimize stress on the breast tissues, it is crucial to gain a comprehensive understanding of the breast movement and deformation patterns.

With the development of motion capture technique (MoCap), the dynamic behavior of breast anatomical landmarks has been captured and analyzed in various studies, confirming that the breast move in complex 3D patterns due to their non-uniform soft-tissue masses [6–8]. However, these studies have 3 intrinsic limitations: (i) MoCap systems rely on physical markers attached to anatomical landmarks on the human body, which limits the number of landmarks that can be tracked; (ii) movements and deformations of unmarked points are not captured or analyzed, i.e. the sophisticated information of the whole surface deformation is discarded; (iii) it remains unclear whether sparse and discrete landmarks are sufficient to fully capture the complexity of breast movement.

On the other hand, three-dimensional (3D) scanning technology can provide information on the entire surface area of the scanned objects and has been applied in anthropometry studies of the human body. Major types of 3D scanning systems are laser based, structure-light based, and multi-view stereo based [9]. All of these techniques require the subject to maintain a specific static position during scanning, after which the surface is reconstructed from the collected signals and exported as mesh data—consisting of an array of vertices, edges and faces to represent the surface topology. With mesh data, post-processing, feature extraction, and measurements [10] can be implemented to extract the geometric information of interest; for example, providing precise and reproducible data for assessing bra fit problems [11–14]. However, while this method provides rich information on the subtle geometric features of the surface, it is limited to static postures and cannot provide dynamic information. This limitation hinders its application in research on dynamic movement and deformation patterns of the breast. Some researchers have attempted to compensate for this limitation by having subjects maintain intermediate postures during scans (from a few seconds to a few minutes) [15–17]. However, this approach presents challenges for the subjects who must hold these postures and may not accurately reflect realistic movements in free motion.

**Fig 1:**
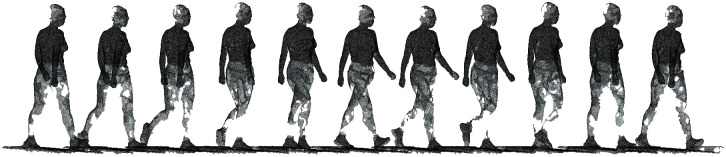
4D scanning mesh sequence recorded in the experiment. From left to right are frames of 0.0s, 0.1s, …, 1.0s.

The development of 3D scanning technology in recent years has made it possible to capture a 3D image within milliseconds, enabling the continuous scanning of human subjects during dynamic activities, i.e. 4D scanning [18]. It extends 3D scanning by adding an extra dimension—time. The output of 4D scanning is typically a sequence of mesh data that represents the scanned surface at different times, as shown in Fig 1. Commercial systems with high scanning rates and accuracy are now available, such as 3dMDbody scanning system (3dMD Ltd., Atlanta, U.S.), which can scan up to 120 frames per second (fps) with an error under 0.7mm (https://3dmd.com/products/#3dmdbody-system-product-specifications). This capability is adequate for capturing subtle deformation patterns of the breasts, and various studies have already begun utilizing this informative modality [19–21]. However, despite the advancement in 4D data collection, there remain two major challenges associated with data processing: (i) the lack of automation schemes: with a scanning rate up to 120 fps (or 7200 frames per minute), manually processing the generated data to extract critical anthropometric measurements is unacceptable. Current research [22–25] usually only extracts a few frames from each scan for processing, leading to discarding subtle information about dynamic procedures and diminishing the value of high-speed scanning capabilities; (ii) the lack of accurate and consistent method to reveal the dense correspondence between frames. While adjacent frames correspond to each other, i.e. the former frame transforms to the next frame, the exact correspondence between vertex points in these frames is unclear. The lack of dense correspondence poses a challenge for developing automated methods to process 4D data, such as tracking the trajectories of anatomical landmarks during dynamic activities.

The estimation of dense correspondence between vertices points of different frames is a core challenge in developing effective methods for 4D data processing. In this paper, we propose a fully-automatic approach named Ulta-dense Motion Capture (UdMC) to reveal such correspondence by augment the sparse landmarks obtained from MoCap as initial dense correspondence and then rectified it with a sophisticated post-alignment scheme. Based on a dynamic human breast anthropometric dataset *DynaBreastLite* constructed in this research, comprehensive estimation of accuracy and efficiency was conducted to evaluate the proposed method’s applicability in dynamic breast deformation pattern research. The results show that the proposed method significantly outperforms the prior baselines in terms of accuracy, consistency, and efficiency. This approach enables tracking, describing, and analyzing dense breast deformation with comprehensively evaluated accuracy, thus providing researchers with an accurate tool to investigate complex breast dynamics during various activities such as exercise or daily movements. To the best of our knowledge, this is the first systematic approach developed for whole-surface dense tracking of breast motion based on 4D scanning data which has significant potential for breast biomechanics, anthropometry, and ergonomics studies, as well as for designing more comfortable and supportive wearable products for female users based on advanced understanding of breast biomechanics.

## Related works

### 4D scanning in anthropometry and ergonomics research

4D scanning systems have been used in ergonomic and anthropometry research to investigate shape changes and deformation patterns during various activities. While the foot [19, 20, 25, 26] and face [21] have received more attention, there is a growing interest in applying 4D scanning technology to analyze body and breast dynamics [22–24]. Although only a limited number of studies have explored this area, it has been recognized as having great potential for ergonomics studies and sportswear design [18]. By capturing rich information on the dynamic changes, interactions, and properties of the human body during active movement that are not available through other modalities, 4D scanning can provide valuable insights into breast dynamics analysis.

However, as has been discussed in the Introduction, the lack of an accurate and consistent method to reveal the *dense correspondence*—which refers to identifying the corresponding points across different frames of scans—has limited the feasibility of developing an automatic data analysis scheme capable of processing the massive volumes of data generated by 4D scanning. This, in turn, has led to acute limitations in applying 4D scanning in anthropometry and ergonomics research. For example, while commercial 4D scanners can scan up to a hundred frames per second, [22, 23] only extracted 3 frames from each gait cycle for analysis, discarding all data of other frames. In another research, [24] proposed a simple automatic analysis scheme that slicing the torso mesh horizontally into 50 layers and then dividing each layer into 360 sectors representing angles ranging from -180° to 180°, with one point extracted from each sector. Points from the same slice of layer and sector are regarded as corresponding points among different frames, and their movement is calculated accordingly. However, [24] admitted that such a scheme may be error-prone since the points with identical height-angle coordinates across different frames are considered to represent the same point on the surface when they may not actually do so.

### Surface matching and registration

In a broader context, the task of identifying dense correspondence between different frames of a 4D mesh sequence falls within the scope of surface registration. This topic has been extensively studied and applied in various fields such as robotic navigation [27], autonomous vehicles [28], augmented reality [29], and medical imaging [30, 31], due to their common needs to reveal the correspondence of two 2D/3D images that are recorded in different times or perspectives.

There are two main types of surface registration methods: rigid and non-rigid [32]. Rigid surface registration is comparatively well-developed, as matching rigid objects is easier than those under free-form deformation. However, this method is not appropriate for breast shape registration due to the highly flexible nature of breast tissue during active movement. For registration of non-rigid surfaces under free-form deformation, various types of approaches have been proposed. Iterative Closest Point (ICP) [33] iteratively matches two point sets to find their optimal alignment, which was then extended to nonrigid registration by [34] and has been adopted by various human scan related studies [10, 35]. Feature-based methods assume that local surface features remain consistent across deformations and utilize such feature to reveal the dense correspondence. However, designing geometric features based on this assumption may not generalize well to highly flexible free-form deformations like breasts. Statistic model-based registration approaches, such as Coherent Point Drift (CPD) [36, 37], model the matching of non-rigid surfaces in a probabilistic fashion and fine-tune the estimated correspondence iteratively. Despite being proposed early on, CPD is still considered one of the state-of-the-art methods [38]. More recently, Bayesian Coherent Point Drift (BCPD) reformulated CPD in a Bayesian setting to improve robustness and accuracy [39].

### Surface registration with auxiliary modalities

Revealing dense correspondence between surfaces remains challenging when dealing with highly flexible surfaces lacking recognizable features, such as human breasts. Previous studies have attempted to address this issue by incorporating information from other modalities [40, 41]. Introducing sparse key points (landmarks) information has been proved to be an effective strategy for solving a wide range of challenging 3D computer vision tasks [42, 43]. [42] constructed a monocular hand 3D reconstruction dataset using a weakly-supervised approach to first detect hand key points and then iteratively fitting them with the deformable 3D model, leading to state-of-the-art 3D reconstruction and pose estimation accuracy at that time. [43] trained a Convolution Neural Network (CNN) that can detect 500+ dense landmarks on the face for head 3D reconstruction. However, these works mainly focus on the 3D reconstruction of a single scene, while our focus is revealing the inter-frame correspondence between different frames within a sequence of reconstructed 3D scenes. In this regard, [44] propose Extended Coherent Point Drift (ECPD), which incorporates sparse prior correspondence information within the CPD framework to provide extra guidance for the registration. Apart from directly introducing prior correspondence, several studies [40, 45, 46] use texture information to provide additional guidance or rectification for registration. FAUST [40] and Dynamic FAUST [46] augment the texture on the body by painting high-frequency patterns on the subject’s skin, resulting in more accurate and robust registration compared to methods that only utilize geometric information [47]. However, these methods require time-consuming and uncomfortable skin preparation before and after scanning. Additionally, Due to the lack of ground-truth data, these methods are evaluated based on some checking criteria [46], which may not be reliable enough for breast anatomical and biomechanics research purposes. Addressing this issue, *DynaBreastLite*, a lightweight dynamic 4D human breast anthropometric dataset was constructed in this research, providing ground-truth anthropometric landmarks trajectories obtained from MoCap.

## Method

### The construction of *DynaBreastLite* dataset

#### Data acquisition

To obtained real-world data of dynamic breast deformation during active motion, we recruited one female participant on December 29, 2021 who completed her participation on the same day. Prior to involvement in the study, written consent was obtained from the participant. The experiment received ethics approval from The Hong Kong Polytechnic University Ethics Committee (HSEAR20210305003). To record the anatomical landmarks for motion model construction, 30 pearl hard base markers were attached to anatomical landmarks around the breasts that could be tracked by the MoCap system as well as recorded by optical cameras and be further aligned to the reconstructed meshes as texture. Comparing with Dynamic FAUST [46] that utilizing painted feature on the skin, attaching markers is comparatively time-efficient and easier to cleanup after scanning. Further more, it can provide accurate ground-truth trajectories for registration accuracy evaluation.

A 3dMDbody scanning system (3dMD Ltd., Atlanta, U.S.) was adopted for 4D scanning, with 30 optical cameras installed around the scanning area to collect images from various perspectives and reconstruct the dynamic surface mesh of the human body based on multi-view stereo. A Vicon motion capture system (Vicon Motion Systems Ltd., Oxford, U.K.) was also installed at the same area to track the landmarks’ spatial trajectories. The scanning rates of 4D scanning and motion capture were set at 120 and 100 fps, which are the highest scanning rates of both systems. During the experiment, the subject was scanned by these two systems simultaneously under two conditions: static standing and 6km/h walking on a treadmill in braless-condition. The static standing data was used to calibrate the two systems to the same coordinates system, as discussed in Alignment of 4D scanning and MoCap data while the later one was used to construct the *DynaBreastLite* dataset. After scanning, the mesh sequence generated by the 3dMDbody system and the landmark-trajectories data were collected for further processing.

#### Alignment of 4D scanning and MoCap data

There are two elements to be considered when aligning the 4D scanning and the MoCap data: the coordinates systems and the recording start times of these systems are different, leading to the requirements of performing spatial and temporal alignment. To align two systems spatially, the static standing data was used since in this case the temporal alignment issue can be neglected:

**Approach**. Spatial alignment of 3dMD and Vicon data

1. Landmark positions was obtained from the first frame of Vicon data and manually labelled from the first frame of 3dMD mesh.
2. In rough alignment stage, axis-rotation *R*_*axis*_ was performed on the Vicon landmarks to oriented them to roughly the same direction as the 3dMD landmarks.
3. In refined alignment stage, the Rigid CPD algorithms [37] was used to estimate the rotation *R*_*fine*_, translation *T*, and scaling *s* to transform the Vicon landmarks to the exact positions of 3dMD landmarks.
4. Eventually, the spatial transformation from the Vicon to 3dMD coordinates system was obtained as:
  $$\begin{array}{c} x_{3dmd}=sR_{fine}R_{axis}x_{vicon}+T \end{array}$$ (1)

For each specific scanning trail, since there may be a small latency between the start times of two systems, it needs to be aligned temporally:

**Approach**. Temporal alignment of 3dMD and Vicon data

1. Reference points extraction For each frame of 3dMD mesh, compute the local gradient of texture gray scale is computed for each vertex:
  $$\begin{array}{c} \Delta_{x}g=\underset{x^{\prime }\in N_{100}\left(x\right)}{\text{max}}\frac{\|g\left(x\right)-g\left(x^{\prime }\right){\|}_{1}}{\|x-x^{\prime }{\|}_{2}+10^{-5}} \end{array}$$ (2)
  where *g*(*x*) is the gray scale value at vertex *x*, $N_{100}\left(x\right)$ is the nearest 100 vertices to *x*. The vertices with local gradient exceeding the mean gradient by more than 2 times of standard deviation are extracted as reference points. Since the texture gray scale level nearby the landmarks changes rapidly, the reference points will contain most of the vertices belonging to the landmarks.
2. The offset *δ* between the two systems defines how to convert the Vicon data timestamp *t*_*vicon*_ to the 3dMD data timestamp *t*_3*dmd*_, i.e. *t*_3*dmd*_ = *t*_*vicon*_ + *δ*. With a specific *δ*, the alignment distance between two systems can be estimated:
  (a) Interpolate the Vicon data as continues trajectories via quadratic interpolation so that it can be resampled at any timestamp during the recording period.
  (b) For each frame of 3dMD mesh, convert its timestamp to *t*_*vicon*_ and resampled the Vicon landmarks positions with this timestamp. The averaged distance between the resampled landmarks and their nearest reference points from the mesh is obtained as the alignment distance for this frame.
  (c) For the whole period, the averaged alignment distance of all frames is obtained as the overall alignment distance.
3. The optimum time offset is obtained by grid search: [−10, 10] is sliced into 100 intervals to obtained the optimum offset *δ*_1_ = −0.30 s, and then the [*δ*_1_ − 0.5, *δ*_2_ + 0.5] is sliced into 100 intervals to obtained the final optimum offset *δ*_2_ = −0.25 s, which already provides a satisfying alignment between the 3dMD and Vicon data, as shown in Fig 3.

**Fig 2:**
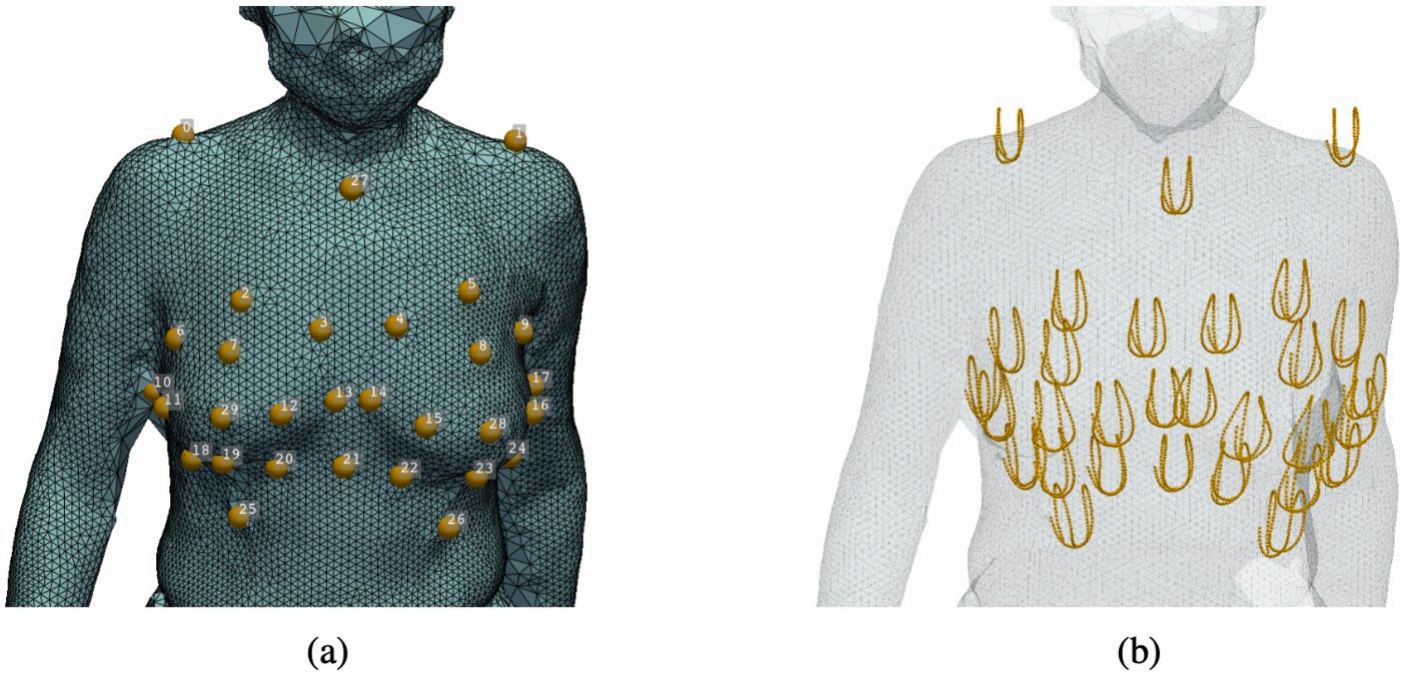
Experiment setting and landmarks setting. (a) the index of the anthropometric landmarks and (b) spatial trajectories of the landmarks.

With the aligned MoCap data as the ground-truth landmark labelling to the 4D scanning data, a lightweight dynamic 4D human breast anthropometric dataset was constructed, referred to as *DynaBreastLite*. The dataset can be accessed via https://huggingface.co/datasets/liu-qilong/dyna-breast-lite. Noted that due to privacy concerns, the texture of the mesh is withheld. This dataset contains 30 anthropometric landmarks in 121 frames of 3D reconstructed scenes, accumulating to 121 frames of 3D meshes and 3630 ground-truth landmark coordinates in total. The data instances in the dataset are referred to using this convention: the *i*-th frame of mesh is denoted as mesh matrix $V_{body}^{\left(i\right)}\in R^{3\times N^{\left(i\right)}}$, where the *j*-th column $v_{j}^{\left(i\right)}$ denotes the 3D coordinates of the *j*-th vertex, with superscript denoting the frame index. The attached landmarks of this frame is denoted as landmark matrix $C^{\left(i\right)}\in R^{3\times K}$, where its *k*-th column $c_{k}^{\left(i\right)}$ denotes the 3D coordinates of the *k*-th landmarks, with subscript denoting the landmark index and superscript denoting the frame index. Noted that the landmarks in different frames with the same landmark index are corresponding with each other, i.e. the landmark $c_{k}^{\left(i\right)}$ is directly corresponding to $c_{k}^{\left(i+1\right)}$ in the next frame. Landmarks index and their spatial trajectories are shown in Fig 2.

#### Automatic breast cropping

To facilitate efficiency of dense breast motion estimation, as a data preprocessing step, the breast area was automatically cropped out based on the contour landmarks of the breasts:

**Approach**. Automatic breast cropping

1. For the *i*-th frame of mesh, the landmarks $c_{0}^{\left(i\right)},c_{1}^{\left(i\right)},c_{10}^{\left(i\right)},c_{17}^{\left(i\right)},c_{25}^{\left(i\right)},c_{26}^{\left(i\right)}$ are selected as breast area contour. The landmark indices is combined as a set *K* = {0, 1, 10, 17, 25, 26}
2. Upper bound cropping
  Calculate the maximum height *h*_*max*_ of all contour landmarks, crop out the mesh part under the level plane at height *h*_*max*_ + *ψ*, where *ψ* is an adjustable factor for slightly enlarging the cropping area. Appropriate *ψ* is empirically selected as 30 mm.
3. Lower bound cropping
  Calculate the minimum height *h*_*min*_ of all contour landmarks, crop out the mesh part above the level plane at height *h*_*min*_ − *ψ*.
4. Estimate the approximate breast plane based on the contour landmarks:
  (a) The center point of all contour landmarks $c_{o}^{\left(i\right)}=\frac{1}{6}\sum_{k\in K}c_{k}^{\left(i\right)}$ is regarded as one point on the breast plane.
  (b) In ideal scenario, all contour landmarks are on the breast plane. In this case, the normal vector *n* of the plane should be perpendicular to $\left(c_{k}^{\left(i\right)}-c_{o}^{\left(i\right)}\right)$, which forms 4 linear equations ${\left(c_{k}^{\left(i\right)}-c_{o}^{\left(i\right)}\right)}^{T}n,k\in K$.
  (c) To exclude 0 vector from the solution space, an extra linear equation ‖*n*‖_1_ = 1 is added.
  (d) In practical scenario, the contour landmarks may not coplane. Therefore, the 5 linear equations are solved with least-square method and then rescale the solution as a unit vector, adopted as the optimized estimation of the norm vector $\hat{n}$. Together with point $c_{o}^{\left(i\right)}$, the breast plane is defined.
  (e) For the sake of geometric completeness, the point $c_{o}^{\left(i\right)}$ is slightly moved towards the inverse direction of the norm vector $\hat{n}$: ${\hat{c}}_{o}=c_{o}^{\left(i\right)}-\psi \hat{n}$. With ${\hat{c}}_{o},\hat{n}$, the breast plane is estimated.
5. Crop the body mesh with the approximate breast plane and removed all disconnected parts. Then the breast area of the mesh is extracted from the body mesh.

**Fig 3:**
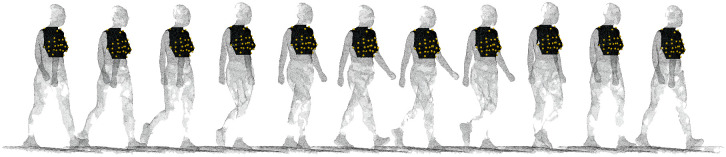
Breast area cropped out based on contour landmarks. From left to right are frames of 0.0s, 0.1s, …, 1.0s.

The *i*-th frame of the cropped out breast is denoted as mesh matrix $V_{breast}^{\left(i\right)}\in R^{3\times N^{\left(i\right)}}$. Automatically cropped out breast area are shown in Fig 3.

### Ultra-dense Motion Capture

**Fig 4:**
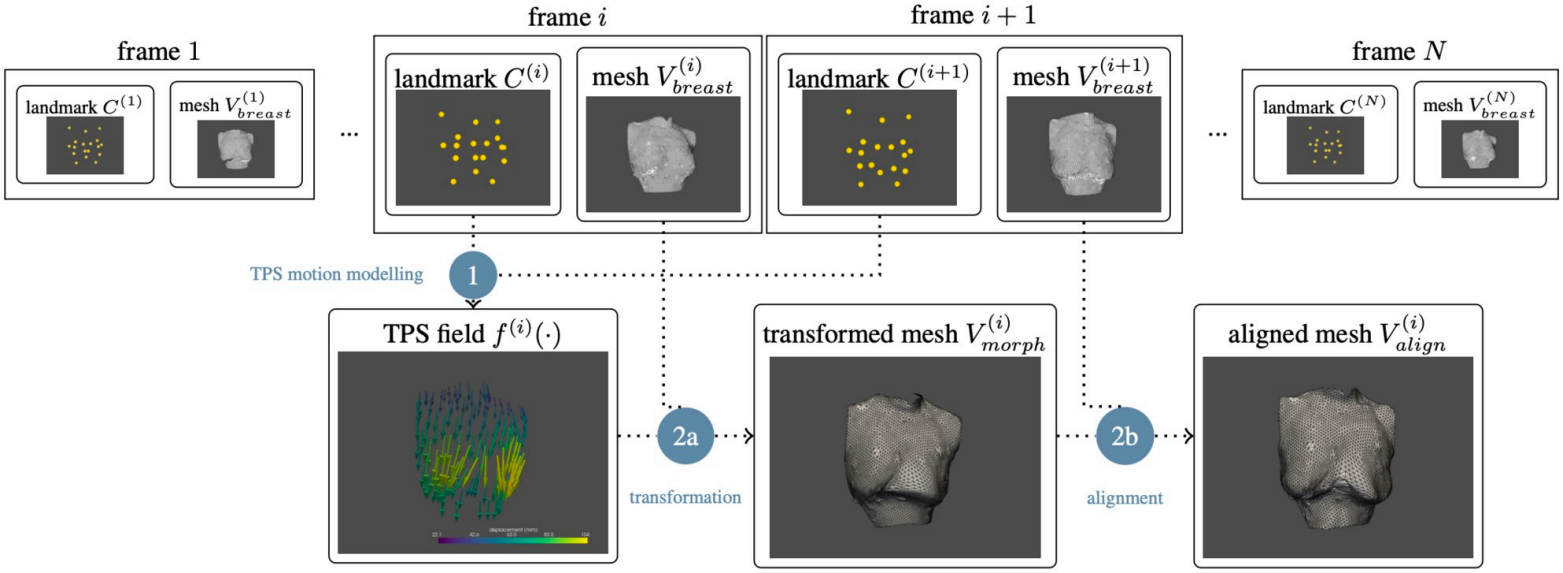
Flowchart of the mesh morphing and post-alignment process. It consists of two major steps: (1) TPS motion modelling based on sparse anatomical landmarks, and (2) post-aligning the transformed mesh to the sophisticated 4D scanned geometry. In step (2), there are two sub-steps: (2a) source mesh transformation and (2b) alignment to the target mesh, which results in an aligned displacement field.

To realistically construct the dense correspondence between different frames of mesh, each frame of breast mesh is morphing to the next frame based on a landmarks guided TPS motion model and a post-alignment scheme, as summarized in Fig 4.

#### Inter-frame mesh morphing and post-alignment

The radial basis function (RBF) is commonly used to interpolate continuous fields from a sparse set of controlling point-value pairs. One type of RBF, known as Thin-plate Spline (TPS) [48], is derived from the physical analogy of bending a thin sheet of metal and offers several advantages: (i) it can estimate a smooth value field with infinite differentiability; (ii) its energy function has a clear physical explanation; and (iii) no manual fine-tuning of free parameters is required. TPS has been widely applied to estimating and describing non-rigid transformations [49, 50]. Considering these advantages, we chose to adopt the TPS kernel for constructing the motion model.

With the sparsely labelled landmarks $c_{k}^{\left(i\right)},k=1,2,\ldots ,K$ in the *i*-th frame as controlling points and their corresponding landmarks in the next frame $c_{k}^{\left(i+1\right)},k=1,2,\ldots ,K$ as the values points, consisting a set of control point—value point pairs, the TPS motion model is constructed. Based on the motion model, an arbitrary point *x*’s corresponding point’s coordinates in the next frame of mesh can be determined as:

$$\begin{array}{c} {\hat{x}}^{\left(i+1\right)}=f^{\left(i\right)}\left(x^{\left(i\right)}\right)=a_{0}+a^{T}x^{\left(i\right)}+\sum_{k=1}^{K}\omega_{i}\phi (\|x^{\left(i\right)}-c_{k}^{\left(i\right)}\left\|\right) \end{array}$$ (3)

where *a*_0_, *a*, *ω*_*i*_, *i* = 1, …, *K* denotes the coefficients of the TPS model, ‖⋅‖ denotes the Euclidean norm, and the function *ϕ*(⋅) denotes a pre-defined kernel function *ϕ*(*r*) = *r*^2^ log *r*. The coefficients of the TPS model are solved with the constraints that inputting the landmarks $c_{k}^{\left(i\right)}$ the corresponding values $c_{k}^{\left(i+1\right)}$ should be the output, which constituting linear equations with close-form solution. Implementation of TPS model and its solution is based on SciPy [51].

With the constructed *f*(⋅), each column of *V*_*breast*_ is transformed to its approximated corresponding coordinates in the next frame of mesh, resulting a mesh matrix $V_{morph}^{\left(i\right)}$. Although the TPS motion model *f*^(*i*)^(⋅) can capture the general trend of movement of the breast based on the labelled anatomical landmarks, it’s only a rough estimation based on sparse landmarks, which doesn’t provide a comprehensive description of the sophisticated breast dynamic characteristic. To introduce more sophisticated dynamic information to the motion model, the 4D scanning sequence is used for post-alignment, i.e. $V_{morph}^{\left(i\right)}$ is further aligned with the target mesh $V_{breast}^{\left(i+1\right)}$: each column of $V_{morph}^{\left(i\right)}$ is replaced by its nearest point from the target mesh based on point-to-plane search [52], resulting in the aligned mesh matrix $V_{align}^{\left(i\right)}$.

#### Continues full-field dense correspondence mapping

With mesh matrix $V_{breast}^{\left(i\right)}$ and $V_{align}^{\left(i\right)}$, the dense correspondence between the *i*-th frame and the *i* + 1-th frame of mesh is constructed. However, it only provide the correspondence of the discrete vertices points. TPS [48] is used for constructing continues full-field dense correspondence mapping:

$$\begin{array}{c} {\hat{x}}^{\left(i+1\right)}=F^{\left(i\right)}\left(x^{\left(i\right)}\right)=a_{0}+a^{T}x^{\left(i\right)}+\sum_{j=1}^{M}\omega_{i}\phi (\|x^{\left(i\right)}-v_{j}^{\left(i\right)}\left\|\right) \end{array}$$ (4)

where $v_{j}^{\left(i\right)},j=1,2,\ldots ,M$ is *M* nearest points of *x* from *V*_*breast*_. Since the point movement should be coherent and smooth, it should be accurate enough to interpolate *x*’s movement based on the neighboring points from *V*_*breast*_. As coefficients *a*_0_, *a*, *ω*_*i*_, *i* = 1, …, *K* is solved with the correspondence pairs constraints, where each correspondence pair constituting one linear equation, such local interpolation scheme is much more efficient than using all correspondence pairs in $V_{breast}^{\left(i\right)},V_{align}^{\left(i\right)}$ for correspondence mapping.

At this stage, the function that maps an arbitrary point *x*^(*i*)^ in the *i*-th frame to its corresponding point ${\hat{x}}^{\left(i+1\right)}$ in the (*i* + 1)-th frame has been constructed. The revelation of the *dense correspondence* in the 4D scanning sequence has paved the way for a comprehensive and systematic analysis of the dynamic characteristics and properties of the breast using 4D scanning data.

### Downstream tasks

#### Virtual landmarks tracking

As discussed in Introduction, the widely adopted MoCap technology relies on physical markers attached to the human body, which limits the number and position of the anatomical landmarks that can be tracked for analysis purpose. The anatomical landmarks of interest must be selected in advance and can not be reselected after a session of recording, leading to numerous duplicated experiments to acquire enough data for analysis. Yet with our approach, the tracking of the movement and trajectory of an arbitrary point on the breast surface can be directly derived from the *dense correspondence* information. This application can be referred to as *virtual landmark tracking*, in contrast with the traditional physical-marker-based landmark tracking approach:

**Approach**. Virtual landmarks tracking

1. Select a virtual landmark *p*^(1)^ from the first frame of breast $V_{breast}^{\left(1\right)}$.
2. Use the dense-correspondence mapping *F*^(1)^(⋅) to estimate its corresponding point ${\hat{p}}^{\left(2\right)}$ in the 2-nd frame of the mesh.
3. With ${\hat{p}}^{\left(2\right)}$, estimate its corresponding point ${\hat{p}}^{\left(3\right)}$ in the 3-rd frame of mesh in the same way, and so on.

#### Deformation intensity analysis

The intensity of deformation of the breasts are associated with discomfort and adverse symptoms of the breasts [5]. Investigating the deformation intensity in different areas of breasts is important not only to comprehend their dynamic characteristics but also to inspire designers in creating comfortable and ergonomic bras. To estimate this information, the trajectory length of anatomical landmarks on the breast is regarded as the metric of deformation intensity of the associated surface-partial. With the result of *virtual landmark tracking*, the deformation intensity of different breast surface-partial can be estimated in fine granularity:

**Approach**. Deformation intensity analysis

1. Evenly sample $V_{breast}^{1}$ as 100 virtual landmarks with quadric decimation [53].
2. Track the trajectory of these virtual landmarks and calculate the trajectory length as a metric for measuring deformation intensity.
3. Coloring each surface-partial as a visual illustration of the estimated deformation intensity.

## Evaluation

### Comparison baselines and implementation

As discussed in Surface matching and registration and Surface registration with auxiliary modalities, probabilistic based surface registration methods, such as CPD [37], have been found to generate comparatively reliable and accurate results. Therefore, we adopt CPD and its more recent variants BCPD [39] from the geometry-only registration approaches as the comparison baselines. Additionally, ECPD [44] is also adopted as baseline due to its similarity with our work in introducing prior correspondence. The implementation of CPD, BCPD, and ECPD algorithms are based on probreg package [54]. However, since we did not augment texture patterns by painting the subject’s skin, texture-based methods such as FAUST [40] and Dynamic FAUST [46] are not included as comparison baselines.

To construct comparison baselines, these methods are used to replace the inter-frame mesh morphing and post-alignment Inter-frame mesh morphing and post-alignment in our approach. Furthermore, since the computation time of CPD, BCPD, and EPCD methods increases drastically when the number of vertices increases, the breast meshes were decimated to 1000 vertices using quadric decimation [53]. The implementation of quadric decimation is based on PyVista [52]. Our approach and comparison baselines were executed on a Dell Precision 3640 Tower workstation (Dell Inc., Round Rock, U.S.A) with Python 3.10.13 and benchmarked on the dynamic human breast anthropometric dataset *DynaBreastLite* constructed in this research. To evaluate the performance of all approaches under different temporal resolutions and 4D sequence lengths, three versions of the dataset were constructed with frame rates of 10 fps, 60 fps, and 120 fps by loading 1 frame from every chunk of 12 frames, 2 frames, and 1 frame from the *DynaBreastLite* dataset, respectively. These sub-datasets are referred to as DBL-10, DBL-60, and DBL-120. The code and dataset can be accessed via https://liu-qilong.github.io/udmc/.

Table 1 summarized the quantitative evaluation metrics of all approaches on all sub-datasets. Results show that our proposed approach outperforms all comparison baselines on all sub-datasets by a large margin. Detailed description and analysis are provided in the following sections.

**Table 1:** Quantitative evaluation metrics.

Dataset	Metric	Ours	ECPD	CPD	BCPD
DBL-10	time (s)	**0.86**	15.38	15.97	60.32
	acc-c (cm)	**0.25**	0.69	–	–
	acc-nc (cm)	**0.33**	5.00	3.08	0.87
DBL-60	time (s)	**5.21**	91.22	90.26	350.12
	acc-c (cm)	**0.36**	1.40	–	–
	acc-nc (cm)	**0.45**	87.60	7.93	1.30
DBL-120	time (s)	**9.32**	183.89	181.88	968.91
	acc-c (cm)	**0.50**	164.78	–	–
	acc-nc (cm)	**0.57**	531.90	9.20	2.24

Metrics are dumped for clarity: *time* refers to computation time, *acc-c* refers to alignment error on control landmarks, and *acc-c* refers to alignment error on non-control landamrks. Noted that *acc-c* metric is not appropriate for CPD and BCPD since they don’t utilize prior-correspondence information during the registration procedure.

### Computation time

**Fig 5:**
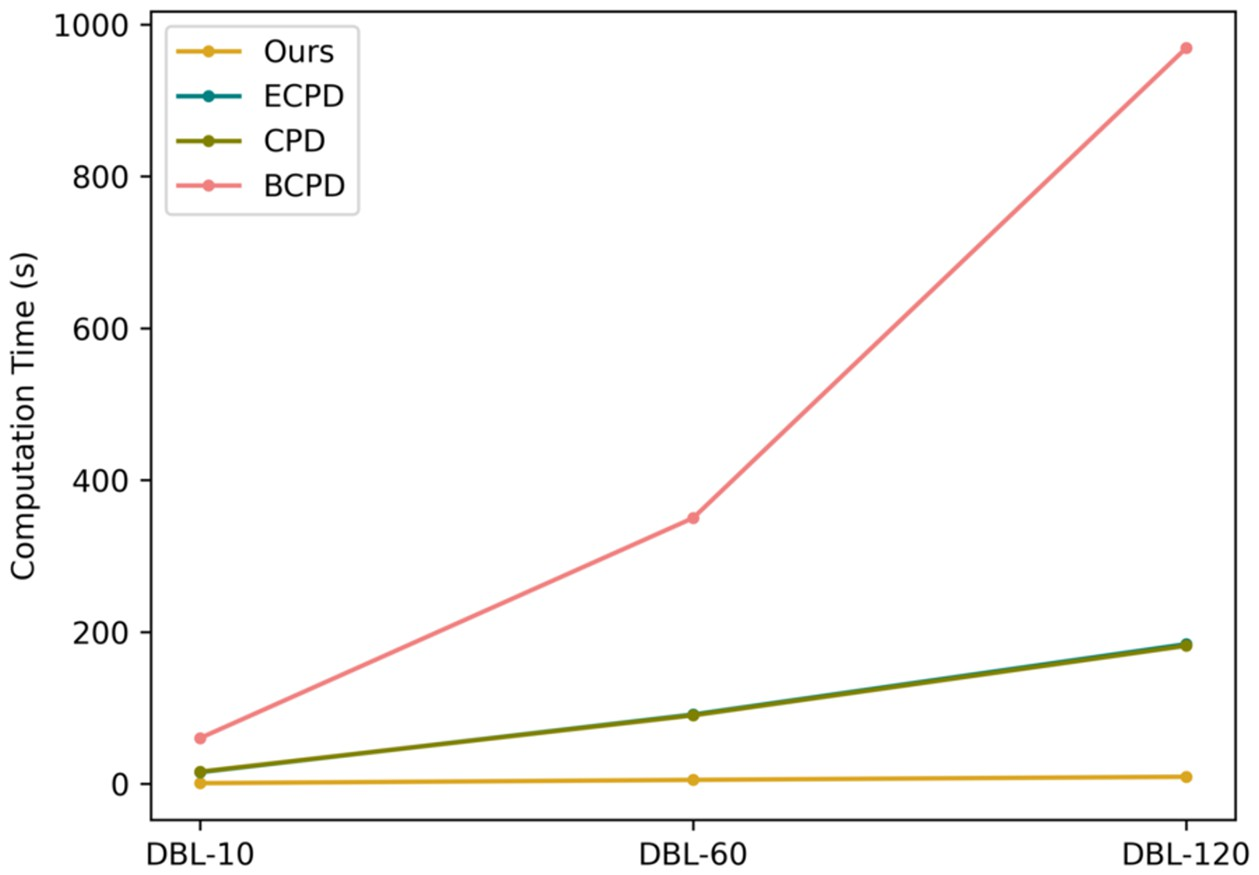
Computation time on all frames of *DynaBreastLite*. Noted that the computation times of CPD and ECPD are so close that their curves overlap with each other.

The computation time was obtained during the implementation of mesh morphing and post-alignment. Data loading, pre-processing stages, and downstream task implementation stages are excluded from computation time evaluation since these steps are identical for all approaches. As presented in Table 1 and Fig 5, for all sub-datasets ranging from 10 fps to 120 fps (sequence length of 11 to 121), our approach has significantly lower computation times of 0.86s on DBL-10, 5.21s on DBL-60, and 9.32s on DBL-120. The advantage in computation time makes our approach more feasible for breast biomechanical and ergonomic studies as well as clinical practice.

### Alignment of control landmarks

Both ECPD and our approach utilize prior-corresponding landmarks (i.e. the control landmarks) as extra information to improve dense correspondence estimation across frames. To estimate their performance in following guidance from prior correspondences, alignment estimation on control landmarks using virtual landmark tracking was conducted:

1. Implement virtual landmark tracking based on initial positions of control landmarks from the first frame of data.
2. Compared trajectories of virtual landmarks with ground-truth control landmark trajectories.
3. Estimated average error (deviation from ground truth) and the standard deviation (SD) of the error.

**Fig 6:**
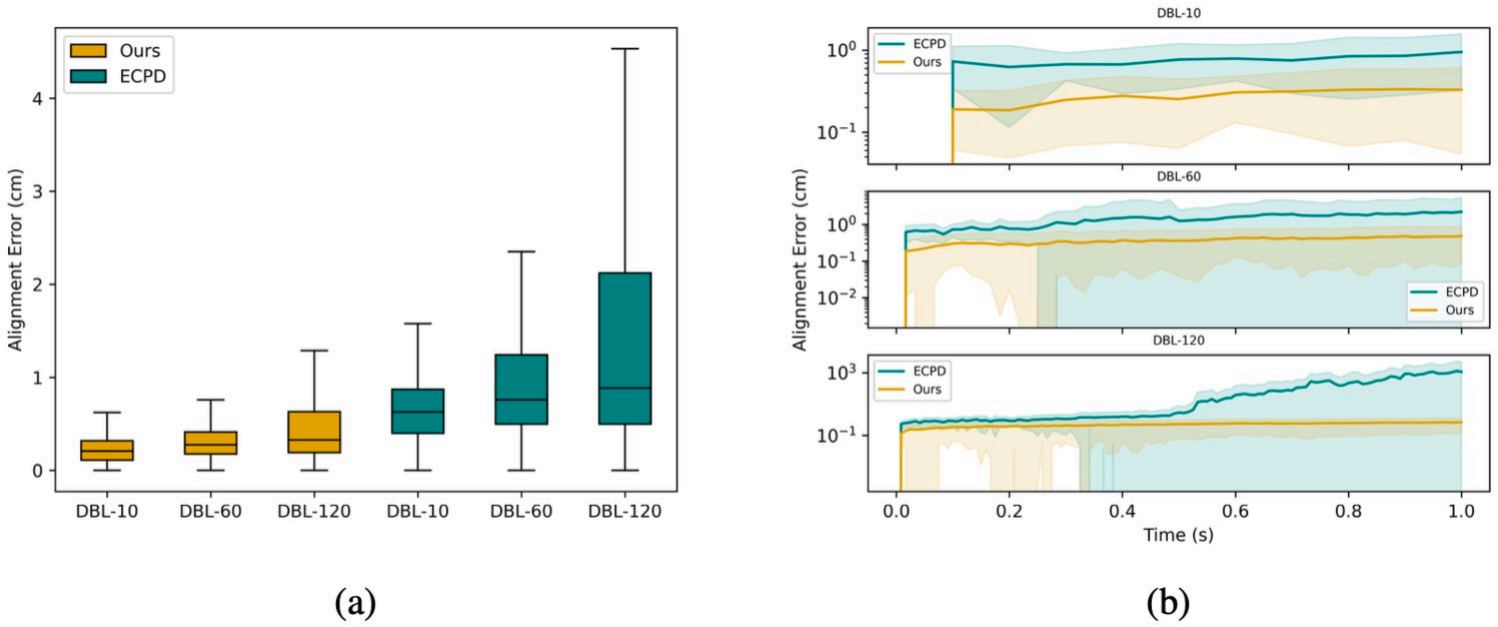
Alignment error on control landmarks. (a) Box plot of overall alignment error: upper/lower boundary of the box represents the third/first quartile of the alignment error; solid middle line represents the median error; the whiskers extend the box by 1.5 IQR; (b) frame-wise alignment error curve. The solid line represents the mean error of that timestamp, while the shaded region denotes one standard deviation above and below the mean, illustrating variability in alignment errors over time. Log scale *y*-axis is used for visual clarity. Noted that CPD and BCPD are neglected from comparison because they don’t utilize prior-correspondence information.

The results are summarized in Table 1 and Fig 6. Our approach achieved the lowest alignment error in all sub-datasets. The frame-wise alignment error curve indicates that our approach aligned more accurately with the control landmarks for all frames. These results demonstrate that our method provides more reliable and consistent alignment.

### Generalization to non-control landmarks

The motion model in our approach is established based on control landmarks, which are specific points used to guide the alignment process. In this case, it is not surprising that our algorithms perform well in aligning these particular points. However, for practical applications, it is essential to estimate the accuracy of alignment on non-control landmark points, i.e. arbitrary anatomical landmarks on the breast. To quantitatively evaluate the accuracy, we conducted leave-one-out validation on all landmarks:

1. Each landmark *c*_*k*_ is excluded one at a time from the dense correspondence estimation procedure.
2. Implement virtual landmark tracking on *c*_*k*_, and compared the tracking result with the ground-truth trajectory of *c*_*k*_. Estimated average error (deviation from ground truth) and the standard deviation (SD) of the error.
3. Since *c*_*k*_ was not included in the dense correspondence estimation procedure, we regarded its estimated error as a sample of non-control landmark tracking accuracy.

For registration approaches that only utilize geometry information (CPD, BCPD), their dense correspondence estimation process does not involve control landmarks; therefore, the differences between virtual landmark tracking results of the labelled landmarks and their ground truth trajectories can be regarded accuracy estimations for non-control landmarks.

**Fig 7:**
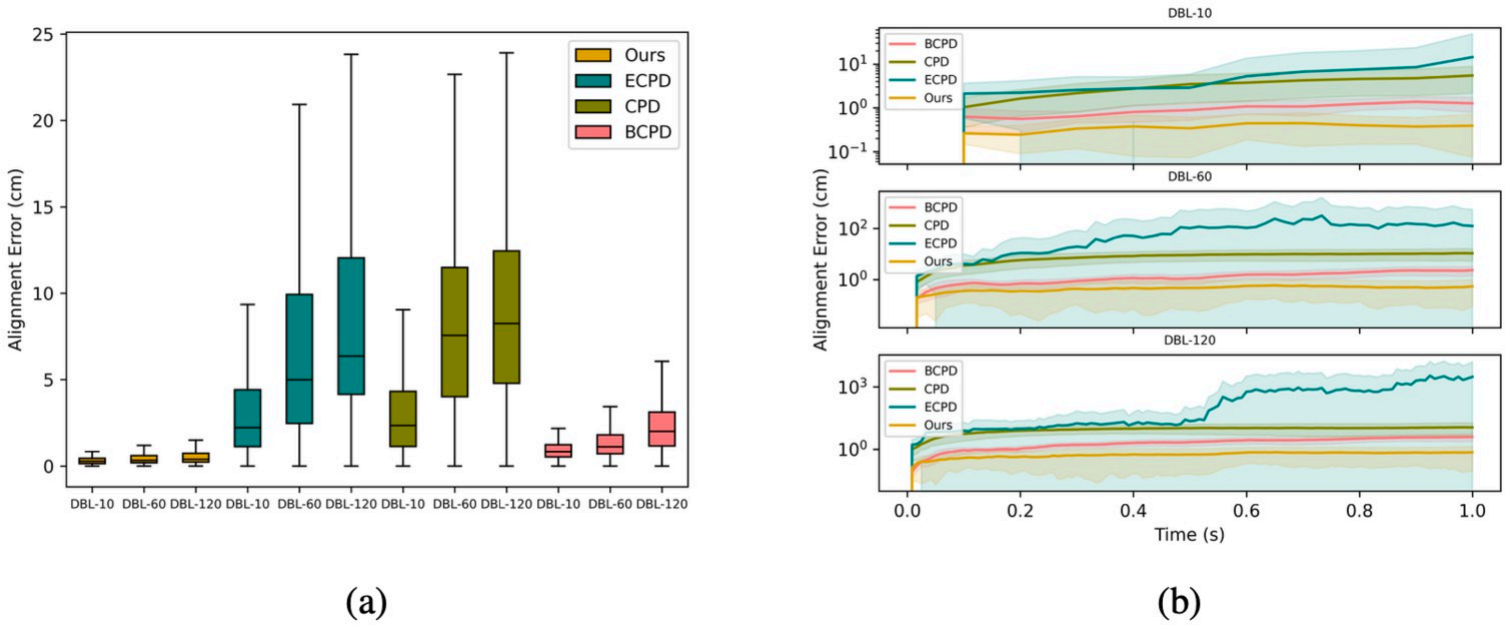
Alignment error on non-control landmarks. (a) Box plot of overall alignment error; (b) frame-wise alignment error curve. Plotting configuration follows Fig 6.

Our approach consistently outperforms the baselines by a large margin, as shown in Table 1 and Fig 7. When the frame rate increases from 10 fps to 120 fps and sequence length from 11 to 121, we observe severe accuracy degradation in all comparison baselines while our approach records a minor acceptable accuracy degradation from 0.33 cm to 0.57 cm, indicating its advantage in scaling with higher frame rates and longer 4D sequences—vital factors for real-world implementation. Besides overall alignment error, it’s also important to consider the frame-wise alignment error curve as it represents the performance and reliability across frames due to accumulated tracking errors. As depicted in Fig 7, error of ECPD accumulates rapidly over successive frames, while other approaches present more stable accumulated errors. Within all approaches, our approach exhibits both the lowest and the most stable error curve throughout the tracking process. This suggests that our method offers greater reliability and robustness for breast motion tracking.

### Downstream tasks

The performance of all approaches on downstream tasks presented in Continues full-field dense correspondence mapping are qualitatively estimated.

**Fig 8:**
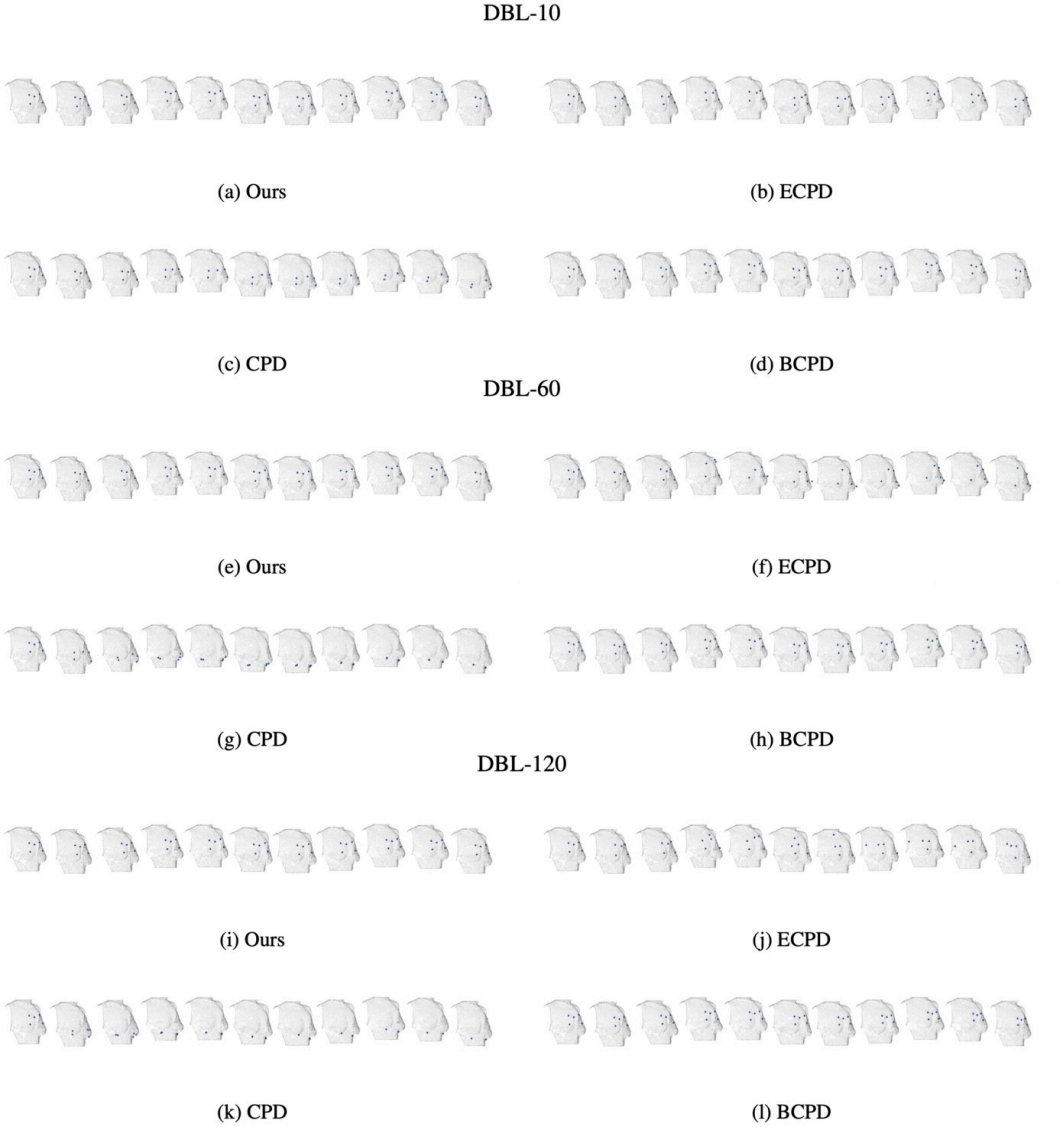
Virtual landmarks tracking results of our approach and baselines. For each plot, from left to right are frames of 0.0s, 0.1s, …, 1.0s.

**Virtual landmarks tracking**. To illustrate the virtual landmarks tracking performance, we selected 5 arbitrary points from the first frame of the mesh as virtual landmarks and tracked their trajectories in the following frames, as shown in Fig 8. Videos of tracking results of all approaches are presented in https://liu-qilong.github.io/udmc/. Noted that the selection of virtual landmarks are merely for visual clarity. Under the hood, every point on the breast surface can be densely tracked based on the continues full-field dense correspondence mapping described in Continues full-field dense correspondence mapping. The results show that CPD tended to sagging all landmarks to lower side and ECPD resulted in a twisted landmark layout during the second half of breast movement especially for higher fps dataset. BCPD and our approach successfully aligned with the swinging motion of breast, but according to Table 1, comparing with our approach, BCPD’s computation time is 70 100 times longer and its alignment error on non-control landmarks is 2.6 3.9 times larger.

**Fig 9:**
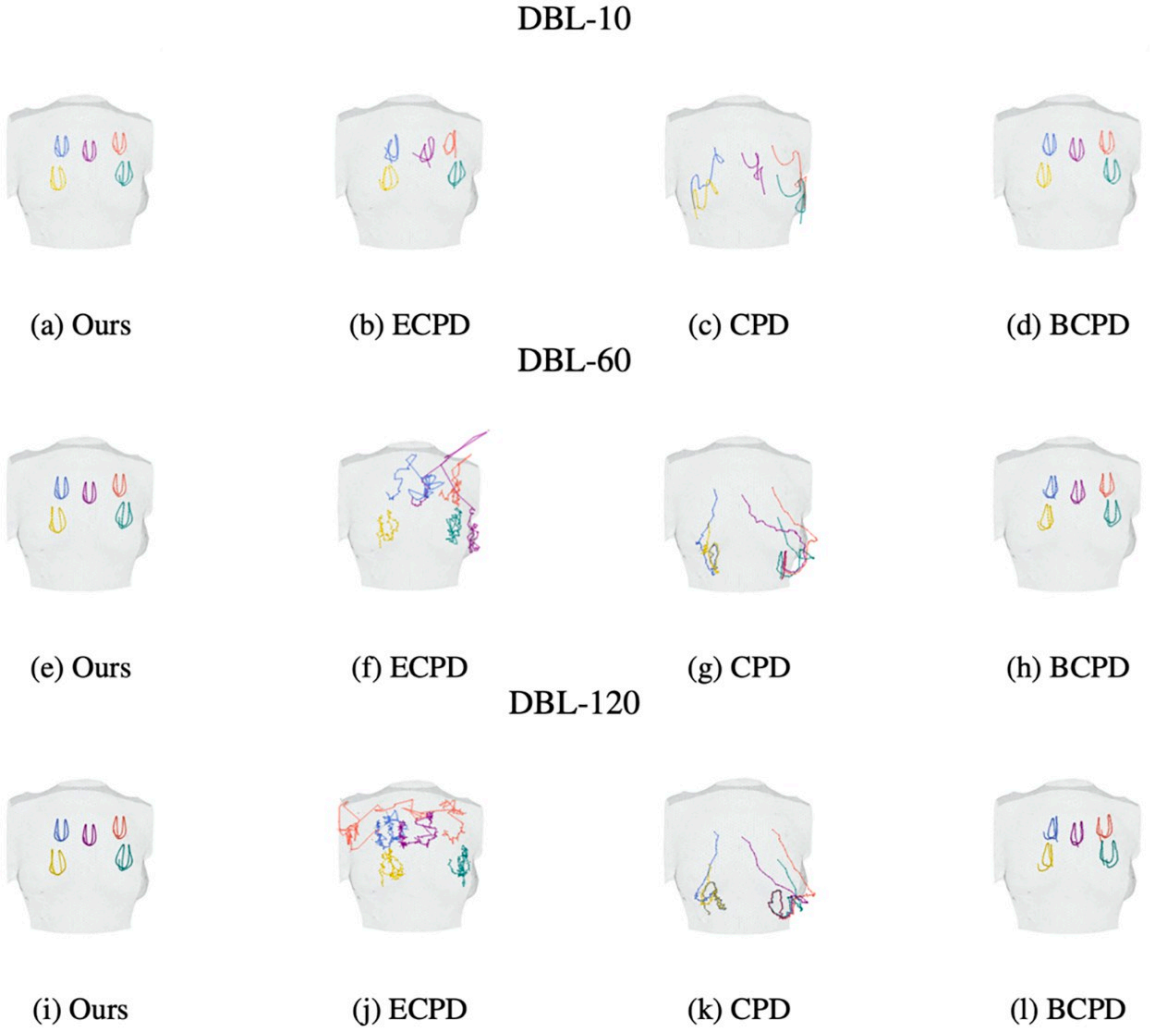
Tracked trajectory of the virtual landmarks.

To illustrate overall breast movement pattern captured by each approach, we plotted continuous trajectories of each virtual landmark as shown in Fig 9. While ECPD and CPD captured chaotic and overlapping trajectories (especially for higher frame rate and sequence length), our approach and BCPD captured smooth butterfly-like trajectories consistent with prior research on breast movement patterns [6]. However, as previously discussed, BCPD requires a significantly longer computation time and records a 2.6 3.9 times larger alignment error. These results suggest that our proposed approach is more suitable for capturing the complex dynamics of breast movement.

**Fig 10:**
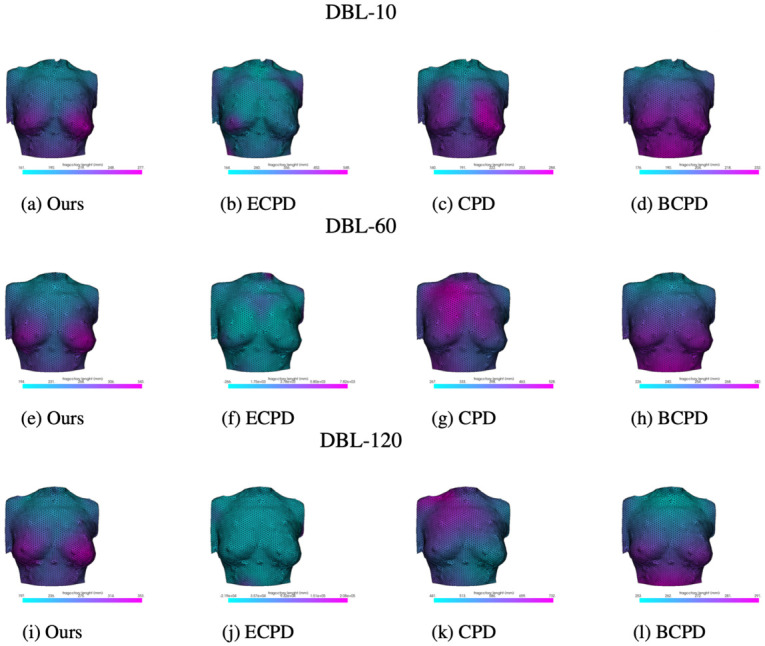
Breast deformation intensity distribution.

**Deformation intensity illustration**. Deformation intensity graphs were generated using all approaches. As shown in Fig 10, ECPD failed to distinguish the differences in deformation intensity around the breast area; CPD and BCPD revealed a smooth increase in deformation intensity from the chest area to nipple areas but failed to identify the differences between the breast soft tissue and the more rigid rib cage area above/beneath the breast; in contrast, our approach captured a smooth and clear boundary in deformation intensity between soft breast tissue and comparatively rigid torso areas, which is consistent with patterns observed in 4D mesh sequences obtained during experiments and prior research on breast deformation patterns [22]. These results show that our approach provides more realistic measurements of deformation intensity.

## Discussion

The proposed approach shows promising results in accurately tracking the breast deformation and providing more optimal results than prior approaches. However, there are still some limitations that need to be addressed: (i) we implement the registration with a simple sequential alignment scheme, which may suffer from accumulated inter-frame error. More sophisticated long & short range alignment techniques [46] may further improve the accuracy; (ii) the *DynaBreastLite* dataset, utilized for evaluation, comprises 30 anthropometric landmarks across 121 frames of 3D reconstructed scenes, accumulating to 3630 ground-truth landmark coordinates in total. However, it is important to note that all the data were collected from a single subject walking at a speed of 6km/h. As an exploratory study, this dataset was constructed for the purpose of validating the proposed method with carefully constructed ground-truth; however, currently it remains limited to the subject/case-specific level. In future studies, we will expand the dataset by recruiting more subjects and covering a wider range of dynamic activities to facilitate data diversity.

Establishing dense correspondence between surfaces is a challenging task that involves determining how points on one surface correspond to points on another surface. Although geometric shape information can provide vital information, it may not be sufficient for entirely solving this problem. For instance, scanning a cylinder rotating along its axis results in identical 3D meshes over time and, therefore, it is impossible to identify rotation movement solely with this kind of scanned geometric shapes. Empirically, as reported in [46], there is a significant gap of accuracy between the geometric-only and the texture incorporated registration schemes. This highlights the importance of introducing other modalities of information when attempting to establish dense correspondence between surfaces. At the core of our proposed approach is the involvement of anatomical landmarks with known inter-frame correspondence relationships. Compared with the geometric-only registration methods that utilize computational intensive iterations to seek for the optimal and coherent point-set alignment, the introduction of prior correspondence guarantees an near-optimal alignment at the beginning, leading to improvement of efficiency and accuracy by a large margin.

Designing an approach to properly and efficiently merge and utilize information from different modalities is challenging. ECPD [44] incorporates sparse prior correspondence information within the CPD framework by multiplying the alignment term of the prior correspondence with the surface alignment term as the objective function, thereby forcing the surface alignment optimization to follow the guidance of the prior correspondence. However, as shown in Evaluation, this approach does not provide better quantitative and qualitative results than state-of-the-art geometric-only approaches like CPD [37] and BCPD [39]. We suspect that this is due to the sparse nature of prior correspondence—alignment term of the prior correspondence contributes much less influence than the surface alignment term. In contrast, our proposed approach utilizes TPS interpolation to establish an initial dense prior-correspondence and then rectifies it with geometric information from a 4D scanning sequence. This technique can be considered an augmentation to previous correspondence information. The evaluation results show that this simple technique can provide a significant performance gain for both accuracy and computation efficiency.

## Conclusion

This study proposes a fully-automatic approach to track the complex deformation of the breasts during dynamic activity with 4D scanning sequence with sparse anatomical landmarks obtained via motion caption (MoCap). A dynamic 4D human breast anthropometric dataset *DynaBreastLite* was constructed in this research and comprehensive evaluation is subsequently conducted, comparing our approach with 3 baseline methods adapted from prior works. Results show that our approach outperforms the comparison baselines by a large margin in terms of accuracy, consistency, and efficiency. For 10 fps dataset, average error of 0.25 cm on control-landmarks and 0.33 cm on non-control (arbitrary) landmarks were achieved, with 17-70 times faster computation time. Two downstream tasks are presented to illustrate its application value: (i) tracking virtual landmarks on arbitrary position without physical markers attached to it and (ii) estimating fine-granularity deformation intensity during activities. Qualitative evaluation shows that our approach can provide more realistic results than other approaches. To validate the proposed approach’s usability on different frame rates and sequences lengths, evaluation was also carried out on 60 fps and 120 fps dataset and consistent and large performance gaining were observed.

The significantly improved performance also suggests that combining 4D scanning sequences and landmarks is a promising approach for constructing a motion model of the human body surface. This also highlights the potential to advance anthropometry studies from the landmarks level to the surface level, thus enabling a more thorough and better understanding of the dynamics deformation patterns and properties of the breasts, with potential to benefit the clinical practices and ergonomic wearable product designs.
